# Transforming Growth Factor-Beta (TGF-β) Signaling in Cancer-A Betrayal Within

**DOI:** 10.3389/fphar.2022.791272

**Published:** 2022-02-28

**Authors:** Abdul Basit Baba, Bilal Rah, Gh. Rasool Bhat, Ifra Mushtaq, Sabra Parveen, Rukhsana Hassan, Mahrukh Hameed Zargar, Dil Afroze

**Affiliations:** Advanced Centre for Human Genetics, Sher-i-Kashmir Institute of Medical Sciences, Srinagar, India

**Keywords:** TGF-β 1, signaling pathways, metastasis, tumor suppressor, tumorigenesis

## Abstract

A ubiquitously expressed cytokine, transforming growth factor-beta (TGF-β) plays a significant role in various ongoing cellular mechanisms. The gain or loss-of-function of TGF-β and its downstream mediators could lead to a plethora of diseases includes tumorigenesis. Specifically, at the early onset of malignancy TGF-β act as tumour suppressor and plays a key role in clearing malignant cells by reducing the cellular proliferation and differentiation thus triggers the process of apoptosis. Subsequently, TGF-β at an advanced stage of malignancy promotes tumorigenesis by augmenting cellular transformation, epithelial-mesenchymal-transition invasion, and metastasis. Besides playing the dual roles, depending upon the stage of malignancy, TGF-β also regulates cell fate through immune and stroma components. This oscillatory role of TGF-β to fight against cancer or act as a traitor to collaborate and crosstalk with other tumorigenic signaling pathways and its betrayal within the cell depends upon the cellular context. Therefore, the current review highlights and understands the dual role of TGF-β under different cellular conditions and its crosstalk with other signaling pathways in modulating cell fate.

## Introduction

What if your confidant turns into a foe? What if a trustworthy becomes a traitor? Does it hold true for transforming growth factor-beta (TGF-β) mediated signaling networks? This review highlights the story of TGF-β signaling and its betrayal within. The exciting story of TGF-β began nearly 4 decades ago, when in 1978, the ground-breaking efforts of De Larco, George Todaro (De Larco and Todaro, 1978) and later in 1981 the work carried out in the Harold Moses and Michael Sporn-Anita Roberts laboratory at the National Cancer Institute (NCI) resulted in the discovery and understanding of TGF-β (Todaro et al., 1981). The early experiments lead to the notion that TGF-β could be a key factor for tumorigenesis. This was based on the ability of TGF-β to “transform” the behaviour of normal fibroblasts forming progressively growing colonies hence the name “transforming” growth factor (Huang et al., 2014). The tumour suppressive role of TGF-β came as another twist when experiments involving epithelial and lymphoid cells showed growth-suppressive effects of TGF-β (Roberts and Wakefield, 2003). Further, evidence suggest that TGF-β promotes the activation of tumor suppressor genes such as *p15*, *p21* and attenuates the tumour promoting gene *c-MYC* expression thereby supports its antitumor effect (Katz et al., 2013). There was a division among the researchers, some believed that TGF-β could be tumour promoter, and some ended up saying that it has a role in tumour suppression. Dysregulation of TGF-β signaling hijacks the complexes of biological functions that plays critical role in developmental processes and tumorigenesis, thus emerges as a promising signaling pathway to be targeted for the anticancer drug development at preclinical and clinical stages (Aashaq et al., 2021). TGF-β signaling pathway has a decisive and dual role in the human cancer progression. Besides promotes apoptosis, cell cycle arrest and autophagy in tumor cells, TGF-β also augments cell stemness, cell motility, angiogenesis, EMT and invasion of tumor cells, suggests that TGF-β plays both tumor supportive and suppressive role (Jena et al., 2021). Thus, TGF-β displays a tumor suppressor phenotype in normal cells and early stages of tumorigenesis, whereas in the later stages of cancer progression, it functions as proto-oncogene and promotes oncogenesis. Cellular signaling pathways are finely interconnected networks which regulate various cellular mechanisms such as cell proliferation and differentiation, embryonic development, angiogenesis, and apoptosis through a series of regulated molecular interactions (Kubiczkova et al., 2012). The complex molecular architecture of signaling pathways is controlled through a defined hub of various protein–protein interactions (Pawson and Warner, 2007). Aberrant alterations of key signaling molecules such as TGF-β could perturb the fine balance of signaling networks thereby leads to the acquisition of hallmark capabilities of cancer (Guo and Wang, 2009). Therefore, the current review highlights and summarizes the recent developments in TGF-β associated tumorigenesis, its antitumor effect as well as cross talks with associated signaling pathways. These findings could resurface new potential therapeutic targets of TGF-β associated signaling pathways in modulating cell fate and could predict new tumor biomarkers for future diagnostics.

### TGF-β Signaling

**FIGURE 1:**
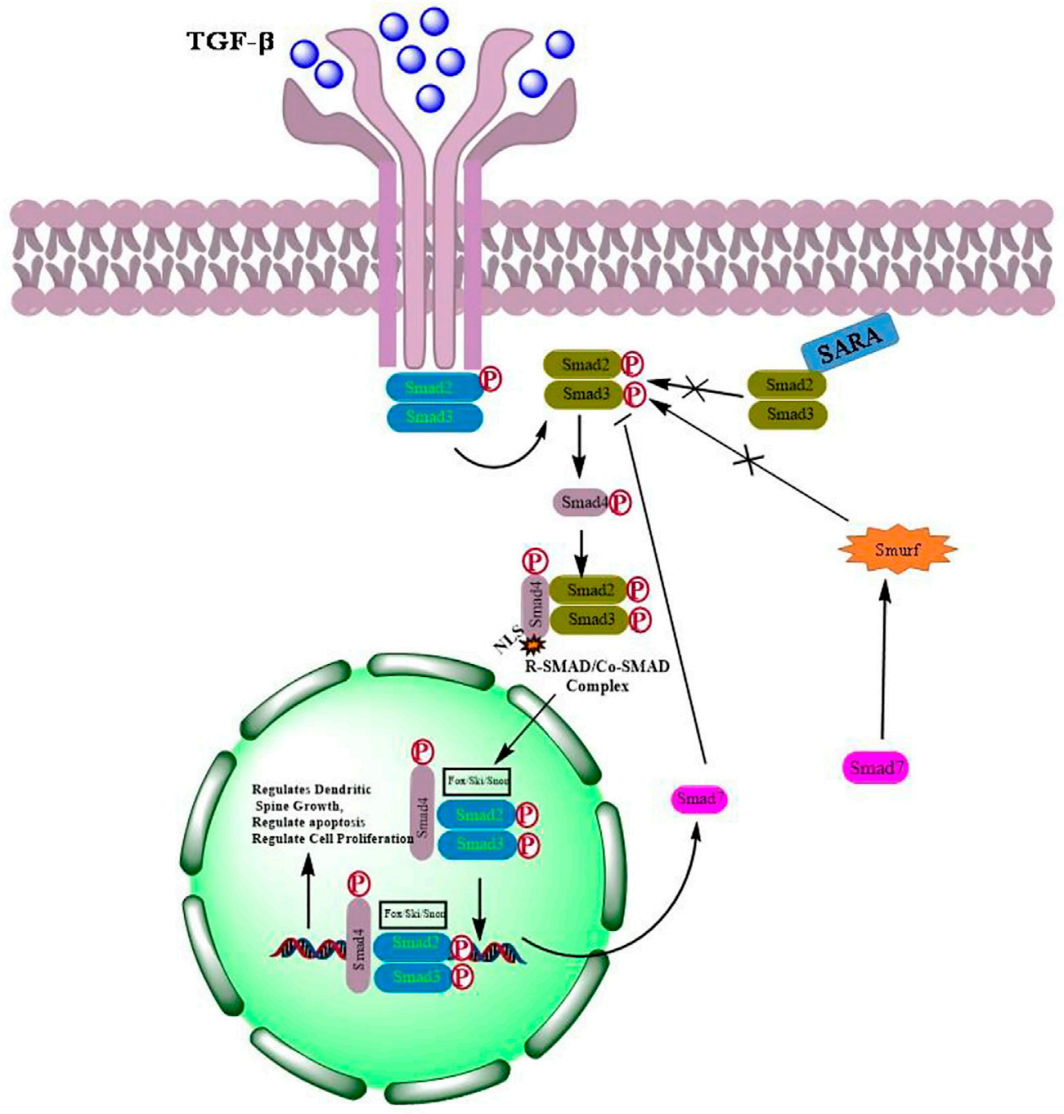
Activation of TGF-β signaling by dimerization of respective receptors followed by phosphorylation and translocation of Smads into the nucleus to regulate transcription of genes involved in cell proliferation, apoptosis and differentiation.

TGF-β, a pleiotropic cytokine, plays a plausible role in a plethora of various physiological processes including growth, differentiation, cell death and migration (Neuzillet et al., 2015; Hata and Chen, 2016). The TGF-β family is further classified into two subfamilies: 1) TGF-β subfamily, which includes TGF-β, activin beta chains, and the protein Nodal, and 2) Bone Morphogenetic Protein (BMP) subfamily that includes BMPs, growth differentiation factors (GDFs), and mullerian inhibitory factor (MIF) (Akhurst and Hata, 2012; Caja and Vannucci, 2015). All these proteins which act as ligands are synthesized as dimeric pre-proprotiens. The pre-proproteins are processed for cleavage by proteases to generate mature functional growth factors which are then finally secreted as latent forms but remains interacted noncovalently with their respective polypeptides (Rossetti et al., 2020). Although, TGF-β activation requires release of active ligands, however, reports suggest that the precursor form of the protein nodal binds to the receptors directly to activate signaling without being processed (Schmierer and Hill, 2007). Mammalian TGF-β ligands exist in three isoforms; TGF-β1, TGF-β2, and TGF-β3. Each of these isoforms binds to their respective transmembrane serine/threonine kinases that bind to type I (TGF-βRI) and type II (TGF-βRII) receptors. Seven TGF-βRI (also known as activin-like receptor kinases {ALKs}, ALK1–7), five TGF-βRII (TGFBR2, BMPR2, ACVR2, ACVR2B, and AMHR2) and two TBRIIIs (betaglycan and endoglin) have been identified so far. Structurally, TGF-β receptors consist of a ligands binding extracellular N-terminal domain, an inner transmembrane region and a C-terminal cytoplasmic serine/threonine kinase domain (Santibañez et al., 2011; Kubiczkova et al., 2012; Hata and Chen, 2016). Binding of TGF-β to the receptors activates signaling via phosphorylation of Smads resulting in the formation of Smad complexes that are translocated to the nucleus where they bind to their respective DNA sequences to regulate the transcription of various target genes (Miyazawa et al., 2002) (Figure 1).

Smads are small intracellular effector proteins which are activated by TGF-β receptors to mediate intracellular TGF-β signaling (Kit Leng Lui et al., 2017). Smads are well conserved and classified into, the receptor-regulated Smads (R-Smads), which include Smad-1, -2, -3, -5 and -8; the common mediator Smad (Co-Smad), Smad-4; and the inhibitory Smads (I-Smads), Smad-6 and -7. R- and Co-Smads are characterized by two highly conserved domains at their N- and C-termini, known as Mad homology domains MH-1 and MH-2, respectively (Wrana and Attisano, 2000). The MH-1 and MH-2 domains are separated by a proline rich and serine/threonine rich linker domain that aids in phosphorylation (Lai, 2001). The linker region also contains phosphorylation sites for mitogen protein kinase and ubiquitin ligase SMURF1 for its recognition (Kamato et al., 2013). Besides, interact with DNA, MH1 domain can also bind with associated proteins which includes transcriptional factors, co-activators, and co-repressors as well as ubiquitination adaptors, and contains a nuclear localization sequence (NLS), whereas MH2 is responsible for oligomerization of Smads, transactivation of Smad nuclear complexes and transcription of key genes involved in various cellular signaling pathways (Xie et al., 2014; Macias et al., 2015; Ahmed et al., 2017). I-Smads have highly conserved C-terminal MH2 domain but lacks the MH1 domain in the N-termini. I-Smads, Smad-6 and Smad-7, function as negative-feedback regulators of TGF-β signaling. Smad-6 prevents the formation of R- and Co-Smad complexes, whereas Smad-7 recruits E3 ubiquitin ligases SMURF1 and SMURF2 for binding to activated TGF-β receptors leading to the ubiquitin-mediated proteasomal degradation (Xie et al., 2014; Tu et al., 2019).

Smad2 is proposed to be a tumor suppressor protein and encoded by the gene present at chromosome 18q21 (Samanta and Datta, 2012). Various malignancies where the mutation rate of Smad2 occurs at a low frequency are non small cell lung carcinoma (NSCLC) 2%, hepatocellular carcinoma (HCC) 3%, colorectal cancer 8% and cervical cancers 8% (Kim et al., 2000). Mutational analysis suggest that majority of mutations of MH1 and MH2 domains of Smad2 are missense mutations, however, in colon cancer Smad2 had two cases of homozygous deletion mutations (Macias et al., 2015). Besides act as a tumor suppressor, Smad2 plays crucial role in development. Smad2 knockout results in early embryonic lethality in mice (Liu et al., 2016). Smad2 missense or homozygous deletion mutations alters phosphorylation, nuclear translocation, and promotes Smad2 auto inhibition thereby leads its degradation. Smad2 is critical for the induction of p21 which regulates cell cycle by acting as a key CDK inhibitor (Moren et al., 2000).

Reports suggest that human tumors have increasing frequency of loss of expression of Smad3 (Vidakovic et al., 2015). Decreased TGF-β responsiveness was observed when Smad3 expression was lost in gastric carcinoma cells, however, TGF-β-mediated tumor suppressor activity was restored when ectopic expression of Smad3 was reintroduced in gastric carcinoma cells, suggests the Smad3 not only acts as tumor suppressor but might also be the target for epigenetic inactivation in gastric carcinoma (Li et al., 2015). Recent evidence suggests that loss of Smad3 expression downregulates TIMP1 expression in choriocarcinoma. This further promotes MMPs activity, thereby plays critical role in tumor invasion (Xu et al., 2003; Rah et al., 2012). Additionally, altered expression of Smad3 is reported to impair the TGF-β-mediated inflammatory response and immune suppression to contribute in tumorigenesis (Hao et al., 2019). Despite Smad3 plays crucial role as a tumor suppressor, recent reports suggest that no embryonic lethality was promoted when Smad3 gene was silenced or knockout. However, it does modulates immune function which later develops colon adenocarcinomas with ability to metastasise to distant secondary sites (Bellam and Pasche, 2010). Another important gene of Smad family located on chromosome 18q is Smad4 (Maru et al., 2004). Smad4 gene is remarkably absent in various cancers such as cervical, prostate, breast, pancreatic, and neuroblastoma due to greater frequency of loss of heterozygosity (LOH) of 18q (Zhao et al., 2018; Rah et al., 2021). The inactivation of Smad4 occurs by various mechanisms which includes frameshift, loss of entire chromosome segment, nonsense and small deletion mutations (Hata et al., 2018). Identified first as deleted in pancreatic carcinoma (DPC-4), Smad4 mutations mainly occurs in pancreatic cancer (Rah et al., 2021). Presence of germ line mutations in MADH4 of juvenile polyposis families further supports that the Smad4 act as a tumor suppressor (Harradine and Akhurst, 2006). Besides, playing critical role in regulating tumorigenesis in various gut associated cancers, Smad4 has been reported to have a crucial role in metastasis (Cheng et al., 2016). Using a cellular and mouse model of TGF-β-induced breast cancer progression, Dekers et al. demonstrated that Smad4 is required for TGF-β induced EMT and bone metastasis of breast cancer cells (Deckers et al., 2006). Further, Smad four knockdown in MDA-MB-231 resulted in the attenuation of EMT transition and bone metastasis thereby highlighting the role of Smad4 in both tumor suppression and progression (Zhang et al., 2015). Collectively, the recent evidences suggest that Smad4 is a key player in regulating tumor progression and tumor suppression depends upon type and stage of malignancy. The inhibitory SMADs (I-SMADs), Smad 6 & 7 with conserved carboxy-terminal MH2 domains regulate TGF-β signaling through a negative feedback mechanism. The I-Smads inhibit TGF-β signaling through interaction with R-Smads and type I receptors. The inhibitory Smads prevent the complex formation of R-Smads and co-Smads. Smad6 particularly inhibits TGF-β signaling by BMP type I receptors ALK-3 and ALK-6 whereas Smad7 inhibits both TGF-β and BMP-induced Smad signaling. SMAD6 and SMAD7 have been shown to play a critical role in tumor progression. Aberrant expression of SMAD6 has been reported in many human cancers. The inhibition of Smad six is known to contribute to the reinstatement of TGF-β homeostasis and is one of the factors for poor survival in patients with NSCLC (Goto et al., 2007). SMAD6 has also been reported to determine the invasiveness of breast cancer cells in BMP-regulated zebrafish xenograft model. SMAD7, first identified in endothelial cells has a conservative Mad homology 2 (MH2) at its C-terminal with no SXSS domain and Mad homology 1 (MH1) domain at N-terminal which is different from the R-Smads and Co-Smads. The feedback inhibition of TGF-β signaling by Smad seven is due to the interaction of L3 loop of MH2 domain and L45 loop of the TGFβR1 kinase domain. In addition to L3 loop, a three finger-like structure in Smad seven provides additional support to bind to TGFβRI (Miyazawa and Miyazono, 2017). The binding of SMAD7 and TGFβRI blocks SMAD2/3 which further prevents the formation of R-Smad/Co-Smad complex, thereby inhibiting core signalling pathway (Pan et al., 2020). In a similar manner, BMP and activin membrane-bound inhibitor (BAMBI) forms BAMBI/SMAD7/TGFβRI complex which inhibits the activation of SMAD3 (Hernandez et al., 2018). Also, a number of proteins can interact with Smad seven to induce the degradation of TGFβRI. For example, the binding of E3 ubiquitin ligase SMAD ubiquitination regulatory factors (Smurfs) to the Smad 7 N-terminal region results in the degradation of TGFβRI (Koganti et al., 2018). In addition to the feedback regulation of TGF-β signaling, Smad7 also interacts with cellular pathways in an independent manner. SMAD7 is known to antagonize Wnt/β-catenin signalling. Smad seven forms complexes with β-catenin/Smurf2 which results in the degradation of β-catenin via proteasome (Vallée et al., 2017). In human prostate cancer cells, the SMAD7/β-catenin interaction plays a crucial role to provoke c-Myc transcription (Tripathi et al., 2019). Smad7 also promotes TNF-induced apoptosis by inhibiting the expression of several anti-apoptotic NF-κB target genes. In addition, Smad7 abrogates NF-κB activity by regulating the activation of TGF-β-activated kinase 1 (TAK1) (Gingery et al., 2008). Smad 7 augments STAT3 activation by directly interacting with the co-repressor gp130, an intracellular domain of leukemia inhibitory factor (LIF) resulting in the disruption of SOCS3-gp130 or SHP2-gp130 complex. Smad7 plays critical role in coordinating gp-130/STAT3 and TGF-β/Smad signalling pathways that promotes pathophysiological processes such as inflammation and tumorigenesis (Yu et al., 2017). Taken together, these findings revealed that I-Smads, Smad 6 and 7 regulate plethora of physiological and pathophysiological processes both TGF-β dependent and independent manner.

### TGF-β as a Tumour Suppressor

TGF-β attains its tumour suppressive role by regulating cell proliferation, apoptosis and immune cell modulation. TGF-β signaling prominently abrogates malignant cell growth through both canonical SMAD-dependent and non-canonical pathway. Through canonical pathway, TGF-β inhibits cell cycle progression through G1-arrest by activating cyclin dependent kinase (CDK) inhibitors p21 and p15. TGF-β suppresses an important oncogene, c-Myc, which stimulates the proliferation and inhibits the transcriptional activation of p21 and p15 (Mukherjee et al., 2010; Katz et al., 2013). In addition, TGF-β inhibits DNA-binding protein inhibitor (ID1, 2, 3) and nuclear factors which plays a crucial role in cell differentiation and progression from G1 to S phase of cell cycle (Katz et al., 2013; Yoshida et al., 2018). TGF-β induces apoptosis in a variety of cell types by modulating the expression of B-cell lymphoma-2 (Bcl-2) family members, death receptor fibroblast associated antigen (FAS), growth arrest and DNA damage-inducible (GADD) 45-β, death-associated kinase (DAPK), and caspases to induce both the intrinsic and extrinsic apoptosis (Zhang et al., 2017). The role of TGF-β as a tumour suppressor has been demonstrated in several cancers (Meulmeester and Ten Dijke, 2011). The non-canonical TGF-β promotes tumor suppressor activity via p38 MAPK pathway to activate caspase-8-dependent programmed cell death. Besides induces tumor suppressive role by activation of programmed cell death, TGF-β promotes tumor suppressive role by regulating immune cell function in favour of tumor cell death (Schrantz et al., 2001). Taken together, TGF-β at the initial stage of tumorigenesis promotes tumor suppression activity, by arresting cell cycle, induces DNA damage and apoptosis is malignant cells.

### TGF-β as a Tumor Promoter

In the later stages of cancer, TGF-β can paradoxically result in tumor progression and metastasis (Katz et al., 2013). Dysregulated expression of TGF-β signaling has been reported in many cancers such as hepatocellular carcinoma, colon, prostate, lung, and breast cancers (Sheen et al., 2013; Zhao and Chen, 2014; Villalba et al., 2017). TGF-β plays an important role in tumorigenesis and promotes tumour development by stimulating epithelial-to-mesenchymal transition (EMT), cell proliferation, invasion, metastasis, angiogenesis and evasion of immune surveillance (Melzer et al., 2017, 2019; Dufour et al., 2018). *In vitro* studies have demonstrated that increase in EMT is associated with the overexpression of Smad-3/4. TGF-β also promotes the secretion of matrix metalloproteases (MMP)-2 and -9, and inhibits the activity of tissue inhibitors of MMPs (TIMPs) (Moustakas and Heldin, 2016; Tan et al., 2017). Collectively, these reports suggest that constitutive activation or dysregulation of TGF-β signalling modulates the expression of various molecules which in turn can promote cell proliferation, invasion, EMT and metastasis to distant sites during late stage malignancies.

### TGF-β as a Therapeutic Target

**FIGURE 2:**
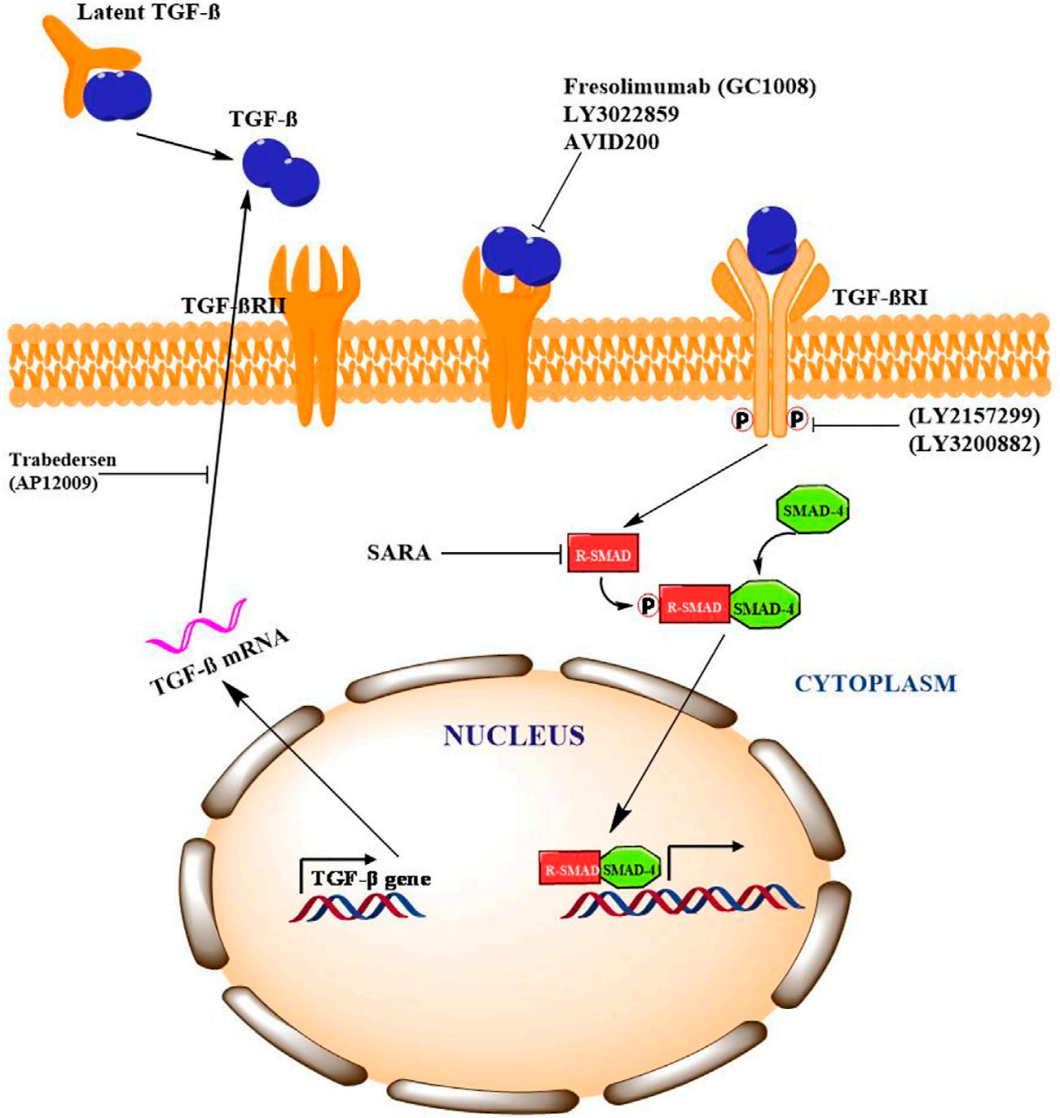
Pharmacological intervention of TFG-β signaling and potential targets Signaling Cross Talk between TGF-β/Smad with other Signaling Pathways.

The complex role of TGF-β in cancer necessitates the comprehensive understanding in order to strategize effective therapeutic approach. A number of pharmacological interventions that target different signaling components of TGF-β have shown promising results in number of preclinical and clinical trials. Different strategies including neutralizing antibodies, ligand trapping, small-molecule inhibitors and antisense oligonucleotides are being explored to target TGF-β signaling. In phase-I clinical trial for malignant melanoma patients, IgG4κ monoclonal antibody, fresolimumab (GC1008), has shown anti-cancer activity by neutralizing TGF-βI, II, and III (Morris et al., 2014). In addition, treating non-small cell lung cancer patients with fresolimumab is still in phase-II clinical trials. Studies in animal models have shown that IgG1 monoclonal antibody, an anti-TGF-βRII (LY3022859) blocks the binding of TGF-β to ectodomain of TGF-βRII which results in significant decrease in tumor growth and metastasis (Zhong et al., 2010). TGF-β ligand trapping by AVID200, a chimeric fusion protein, prevents binding of TGF-β to the receptor. *In vivo* study by Sanjabi et al., demonstrated that AVID200 enhanced the anti-cancer activity in immunocompetent host mice (Sanjabi et al., 2017). AVID200 is currently in phase-I clinical trials for advanced solid tumor patients (Yap et al., 2020). Galunisertib (LY2157299), a small-molecule inhibitor, binds to TGF-βRI thereby inhibiting its kinase activity. Preclinical study by Yingling et al., in *in-vitro* and *in-vivo* models demonstrated anti-tumour activity of galunisertib (Yingling et al., 2018). Phase-I clinical trials of galunisertib revealed promising anti-cancer activity in patients with pancreatic cancer, glioma, HCC and advanced solid tumours (Fujiwara et al., 2015; Ikeda et al., 2019; Wick et al., 2020). LY3200882, a potent ATP-competitive TGF-βRI inhibitor has shown antitumor activity in both preclinical mouse model of TNBC as well as patients with metastatic cancers (Pei et al., 2017). Another strategy is antisense oligonucleotides (AON) which are specifically designed to block the translation of genes. Trabedersen (AP12009), an AON, targeting TGF-βRII mRNA has shown promising effects in phase-I clinical trials for patients with pancreatic cancer, colorectal cancer and melanoma (Oettle et al., 2011). Nemunaitis et al., has shown that Belagenpumatucel-L or Lucanix, an AON vaccine targeting TGF-βRII improved the overall survival of NSCLC patients after chemotherapy (Nemunaitis et al., 2009). Collectively, these evidences indicate that TGF-β is a promising therapeutic target to inhibit tumorigenesis in plethora of cancers as described in Figure 2.

### PI3K/Akt Signaling

The PI3K/Akt signaling pathway is a master regulator of various physiological and cellular processes including cell proliferation, growth, and survival (Rodgers et al., 2017). PI3Ks are classified into three classes based on the structure, distribution, substrate specificity and mechanism of action. PI3Ks are phospholipid kinases, existing as a heterodimer of a regulatory subunit p85 (p85α, p85β, p55α, p55γ and p50α) and a catalytic subunit p110 (p110α, p110β, p110γ, and p110δ) (Piddock et al., 2017; Giordano and Kiger, 2020). The multiple receptor tyrosine kinases (RTKs) or G-protein-coupled receptors (GPCRs) activate PI3K which inturn phosphorylate phosphatidylinositol 4,5-bisphosphate to form phosphatidylinositol 3,4,5-trisphosphate (PIP3) (Mantamadiotis, 2017). PIP3 binds to the pleckstrin homology (PH) domains of various signaling proteins, including phosphoinositide-dependent kinases (PDK1) and its downstream target protein kinase B/Akt (Krygowska and Castellano, 2018; Gesmundo et al., 2019). The phosphorylation of the two critical amino acid residues, Thr308 and Ser473 is essential for full Akt activation (Yu and Cui, 2016). Akt has three isoforms: Akt1, Akt2 and Akt3, that are expressed from distinct genes located on separate chromosomes (Rahmani et al., 2020). Akt1 and Akt2 are ubiquitously expressed in human tissues, while Akt3 is restricted to brain and testes (Ji et al., 2015; Tian et al., 2019).

**FIGURE 3:**
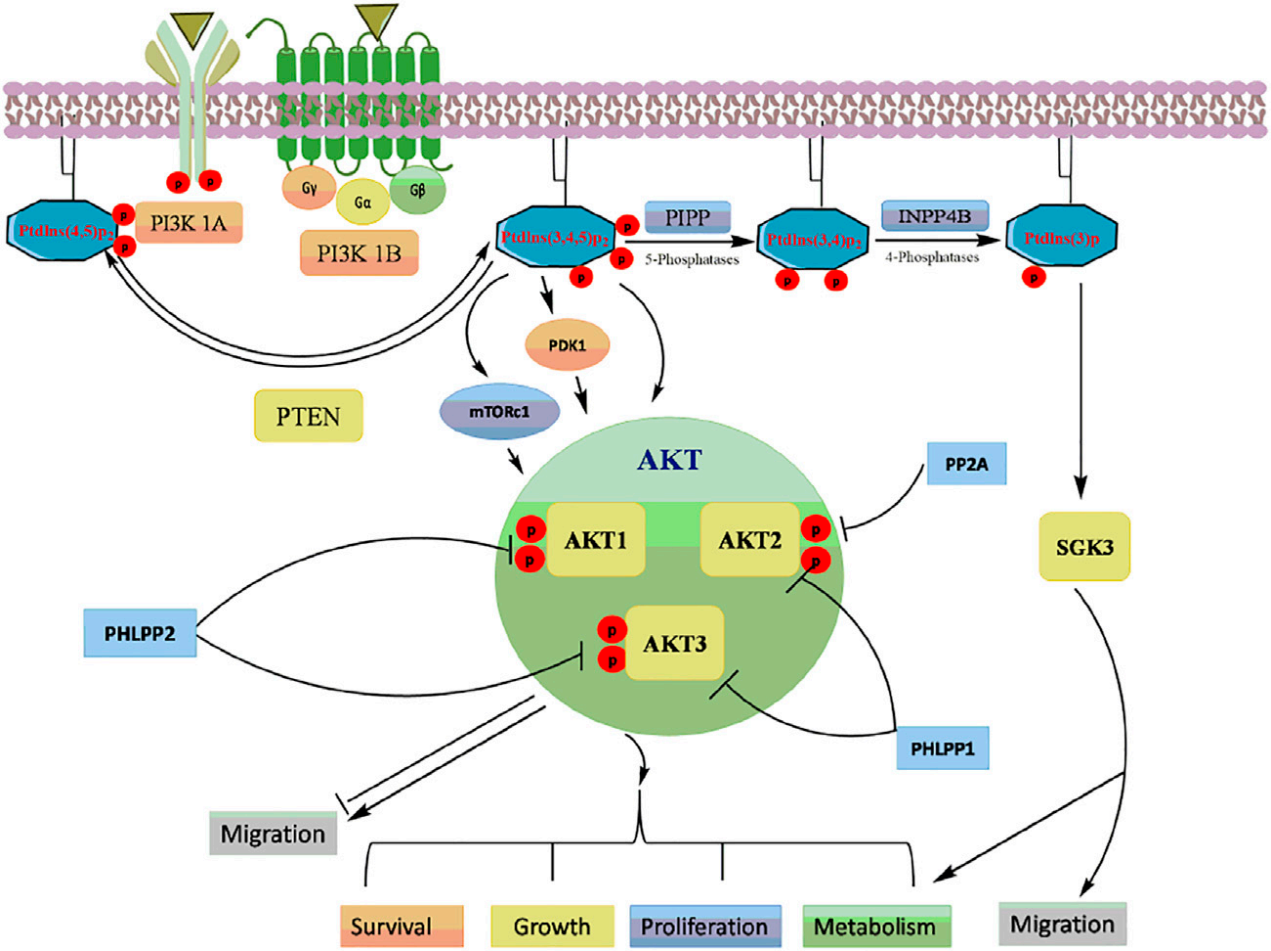
A schematic representation of the PI3K/Akt signalling and its downstream targets.

Akt activation causes the phosphorylation of many downstream targets in the cytoplasm and nucleus, explaining its relatively broad range of downstream effects and increases cell proliferation, invasion, and angiogenesis (Hinz and Jücker, 2019) (Figure 3). Activated Akt inturn phosphorylated wide range of target proteins including glycogen synthase kinase-3β (GSK-3β) (Hinds et al., 2016), forkhead box O transcription factor (FOXO) (Norambuena-Soto et al., 2017), Mouse double minute two homolog (MDM2) (Li et al., 2020), inhibitor of IkB kinase (IKK) (Ghoneum and Said, 2019), Bcl-2 interacting mediated cell death (BIM) (Kapoor et al., 2020), Bcl-2 associated agonist of cell death (BID) and Bcl-2 associated X protein (Bax) (Liu et al., 2020). The PI3K/Akt pathway is tightly regulated by lipid phosphatase enzyme phosphatase and tensin homolog (PTEN), which negatively regulates the kinase activity of PI3K (Haddadi et al., 2018).

Hyperactivation of the PI3K/Akt pathway is frequently seen in many cancers (Rasool et al., 2017; Rodgers et al., 2017). PI3K/Akt can activate NF-kB signaling by phosphorylation IKK or by stimulating nuclear translocation of NF-kB (p65) thereby inducing cell proliferation and apoptosis evasion (Tilborghs et al., 2017). Akt is known to inhibit proapoptotic proteins such as Bax, Bad and procaspase-9 (Wang et al., 2018). Akt also antagonizes p53-mediated apoptosis by phosphorylation MDM2 contributing to chromosome instability in cancer (Cao et al., 2020). Several studies have documented an increase in the expression of PI3K and Akt with suppression of PTEN in various human cancers (Han et al., 2018). Recent evidences suggest that PI3K/Akt pathway has been extensively linked with TGF-β signaling pathway majorly in stem cells and tumor cells of various tissues (Yeh et al., 2018). Although the cross-talk of these pathways is intricated, mutual regulation depends upon cellular context and associated pathophysiological processes. Depending on cellular context this crosstalk can result in either inhibition or activation of various downstream molecules critical for biological processes. TGF-β can directly or indirectly activate PI3K-Akt pathway. The key components of TGF-β family Smad2/3 activation in coordination with hyperactivation of PI3K, modulates cell fate of human embryonic stem cells (hESCs) by upregulated the expression of Nanog which is a key pluripotent gene essential for self renewal (Gordeeva, 2019). Moreover, increased expression of PI3K inhibits ERK/MAPK signalling which results in GSK 3B activation leading to b-catenin inhibition. Since Smad2/3 in association with b-catenin is required for mesendoderm gene expression initiation, low PI3K activity allows β-catenin and Smad2/3 complex for direct mesoderm differentiation (Jason et al., 2015). In epithelial and lymphoid cells, smad dependent TGF-β signalling in known to inhibit cell proliferation and induces apoptosis in hepatocytes and resting B cells. PI3K/Akt signaling antagonizes TGF-β-mediated proapoptotic effect in B cells and hepatocytes by allowing interaction of Akt with Smad3 in cellular milieu. The binding of Akt to smad three results in sequestering smad3 which prevents Smad3 dependent apoptosis in hepatocytes (Papoutsoglou et al., 2019). Another study suggests that the cytostatic effect of TGF-β/Smad3 signaling is promoted via Akt-mediated phosphorylation of FOXO. The phosphorylated FOXO interacts with Smad3 to block its translocation into the nucleus thereby preventing transcription of genes involved in apoptosis (Yadav et al., 2018). This inhibition promoted by PI3K/Akt signaling switches the role of TGF-β from tumor suppression in early tumorigenesis to tumor promotion in the late stage tumorigenesis (Syed, 2016). Additionally, PI3K/Akt pathway in coordination with TGF-β signaling regulates EMT, cell invasion, and metastasis in various types of malignant cells (Luo, 2017). TGF-β phosphorylated Akt at Ser-473 and activates its kinase activity via integrin-linked kinase (ILK) (Tsirtsaki and Gkretsi, 2020). This activation promotes optimal transcriptional activity of Smad3 to upregulated expression collagen I in mesangial cells. A mechanistic study by Runyan et al. has demonstrated that PI3K/AKT signalling influences the expression of collagen I in mesangial cells stimulated by TGF-β (Runyan et al., 2004). Cancer cells in the tumor microenvironment require enhanced glycolysis to survive and proliferate. In glioblastoma cells, Smad dependent TGF-β signalling is known to target p38 MAPK and PI3K/Akt signaling pathway which in turn increases the expression of PFKFB3 and induces glycolysis (Rodríguez-García et al., 2017). Also, in normal murine mammary gland epithelial cells, TGF-β promotes the expression of connexin43 gene expression by activation p38 and PI3K/AKT signaling (Tacheau et al., 2008).

Together, these evidences suggest that PI3K/Akt signaling is linked with TGF-β signaling at multiple crosstalk points during tumor development. Depends upon the cellular context and influence of other signaling pathways, TGF-β could act as tumor suppressor by promotes apoptosis and/or tumorigenic regulates critical events such as EMT, invasion and metastasis of malignant cells (Figure 7).

### NF-kB Signaling

NF-kB was first discovered as a transcription factor in the nucleus of B cells where it was reported to bind to the enhancer region of the k-light chain of immunoglobulin family. The NF-kB proteins are divided into two subfamilies, the ‘NF-kB’ proteins (p50/NF-kB1, and p52/NF-kB2) and the ‘Rel’ proteins (RelA/p65, c-Rel, RelB) (Tilborghs et al., 2017; Mitchell et al., 2019). These proteins are characterized by a highly conserved domain (Rel homology domain) of 300 amino acid residues essential for homo- or heterodimer formation to interact with DNA and IkB family of proteins (Kanapeckaitė et al., 2021). The C-terminal region of the RHD has a nuclear localisation signals that helps in the delivery of active form of NF-kB complexes into the nucleus, whereas the N-terminal region contains the DNA-binding domain (Serasanambati and Chilakapati, 2016). In addition, Rel proteins comprises of a transactivation domain (TAD) at C-terminal whereas NF-kB subfamily members contains multiple copies of ankyrin repeats which act to auto-inhibit these proteins (Collins et al., 2016). The activation of NF-kB can occur by two separate pathways, classical (canonical) or non-classical (non-canonical or alternate) pathway (Ichikawa et al., 2015; Park and Hong, 2016).

*The Canonical Pathway:* This pathway is activated primarily in response to many internal factors including tumor necrosis factor-alpha (TNF-α), interleukin (IL)-1β, epidermal growth factor (EGF), T- and B-cell mitogen, bacteria, and lipopolysaccharides, viral proteins, double-stranded RNA, and external agents involving physical and chemical stress (Chen et al., 2018a; Taniguchi and Karin, 2018). Initially NF-kB is inactive in naive cells that are not yet stimulated by external signals and the p50/p65 heterodimer is retained in the cytosol by inhibitor protein, IkB (Ghafoori et al., 2009). The IkB family consisting of IkB-α, IkB-β and IkB-ε subunits comprises of six ankyrin repeats that prevents the translocation of p50/p65 into the nucleus by shedding the activity of nuclear localisation signals of NF-kB (Morotti et al., 2017). The activity of IkB is tightly regulated by IKK, a large multisubunit kinase complex consisting of two kinase subunits, IKKα (IKK1) and IKKβ (IKK2), and a regulatory subunit IKKε (NEMO). In response to NF-kB inducing signals, both IKKα and IKKβ induce phosphorylation and degradation of IKB proteins (Galluzzi et al., 2012). The disintegration of IKB leads to the release and subsequent translocation of NFkB p65-p50 heterodimer into the nucleus, where it binds to the kB elements to mediate the transcription of responsive genes involved in cell growth, differentiation and survival, immune response, inflammation, apoptosis, invasion, metastasis, and angiogenesis (Yuan et al., 2016; Sun, 2017; Taniguchi and Karin, 2018).

**FIGURE 4:**
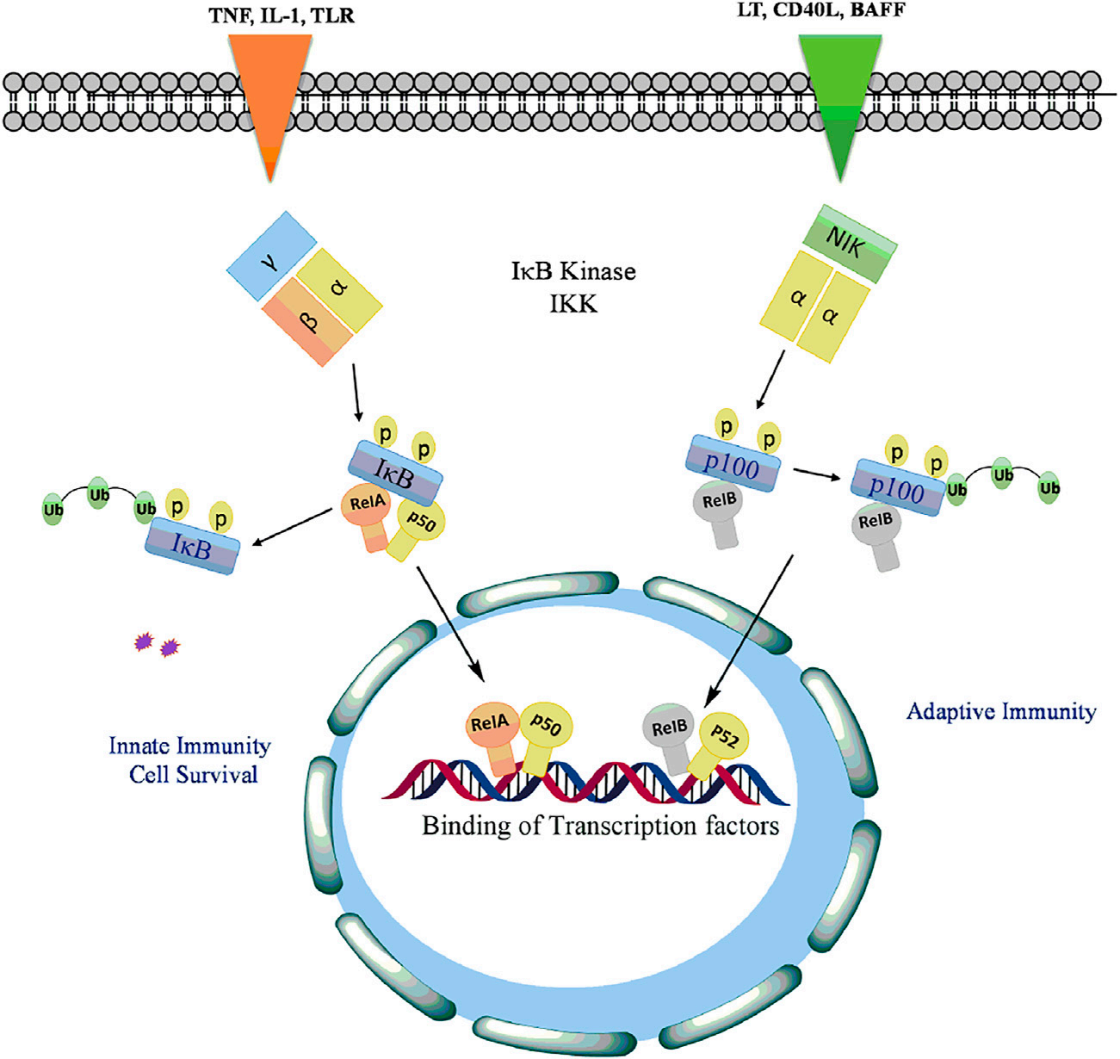
Activation of NF-kB signaling by phosphorylation of IkB with TLRs and proinflammatory cytokines to release and allow translocation of RelA, p50, RelB into the nucleus to regulate transcription of genes involved in cell proliferation, antiapoptotsis, inflammation, cell survival, innate and adaptive immunity.

*The Non-canonical Pathway:* Various members of TNF cytokine family such as lymphotoxin, B-cell activating factor belonging to the TNF family (BAFF), CD40 ligands or viruses such as Epstein-Barr virus (EBV) and T-cell leukaemia virus (Zhang et al., 2020b) activates the non-canonical pathway of or alternative NF-kB signaling pathway. This pathway involves phosphorylation and activation of IKKα by the NF-kB-inducing kinase (NIK) which in turn phosphorylated NF-kB2 (p52/p100) at Ser866 and Ser870 (Demchenko et al., 2014). The phosphorylation of p52/p100 by IKKα results in the proteasomal degradation of p100 leading to activation of RelB/p52 heterodimer (Roy et al., 2018). The active p52-RelB heterodimer translocated into the nucleus binds to respective elements and regulates the expression of genes required for lymph organogenesis and B-cell activation (Park and Hong, 2016) as depicted in Figure 4.

**FIGURE 5:**
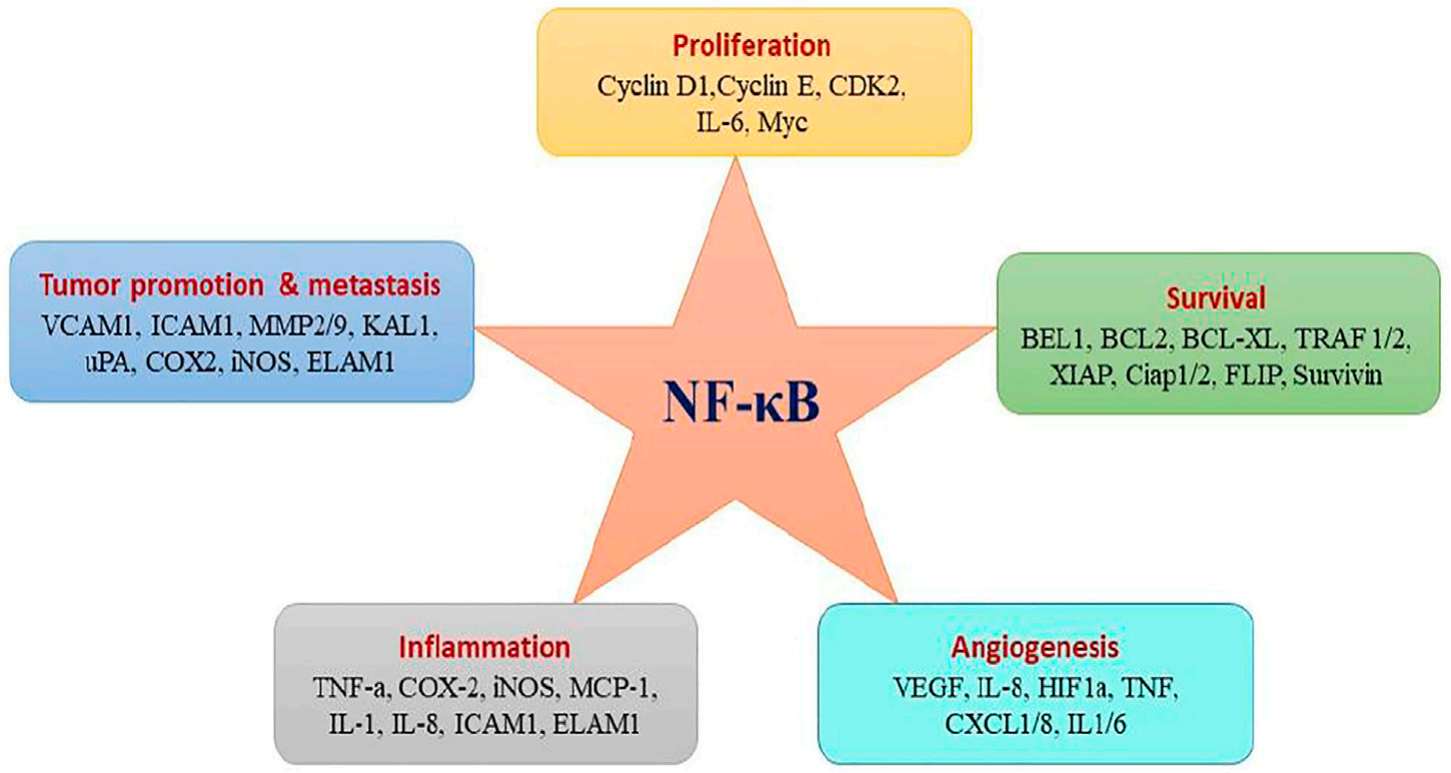
NF-kB activation affects hallmarks of cancer trough the transcription of genes involved in cell proliferation, survival, and angiogenesis.

Several *in vitro* and *in vivo* studies have revealed the constitutive activation of NF-kB and its association with many types of cancers including breast, lung, colon, pancreas, head and neck, oesophagus as well as melanomas, lymphomas (Xiao et al., 2018), and its role has been associated with various tumour-favouring cellular processes including cancer cell proliferation, preventing apoptosis, and increasing a tumor’s angiogenic and metastatic potential (Park and Hong, 2016). Figure 5 shows the various target genes of NF-kB. NF-kB is activated in cancer either from extrinsic signals in the tumor microenvironment or from intrinsic deregulation of the pathway within the tumor (Lee et al., 2017). Various factors such as autocrine secretion of inflammatory mediators (chemokines and cytokines), mutations and/or overexpression of ligands and receptors (EGF, hepatocytes growth factors and integrins), activation of kinases (IKK, NIK, GSK-3β, Akt/PKB, and mutation with defective function of IkB-α contribute to constitutive activation of NF-kB (Nagel et al., 2015; Lee et al., 2017).

Aberrant activation of TGF-β in association with NF-kB signaling has been documented in various cancers (Zappavigna et al., 2020). Activation of NF-kB by TGF-β has been reported to mediate the transcriptional activation of various TGF-β target genes (Torrealba et al., 2019). A study by Kon et al. (Khafaga et al., 2019), has shown that TGF-β triggers TNF-α or interleukin-1 to activate type VII collagen gene expression through NF-kB-binding site and SBE sites in various regulatory gene sequences (De La Cuesta et al., 2019). Aberrant activation of TGF-β/NF-kB signaling pathways has been documented to promote EMT and angiogenesis. TGF-β activates transcription of NF-kB target genes and promotes EMT in pancreatic cells as well as proliferation and differentiation of keratinocytes (Khatami, 2017). Activation of the NF-kB by TGF-β can be mediated by both canonical Smad pathway and non-canonical Smad pathway (Tripathi et al., 2019). In Canonical Smad pathway, Smad3 is shown to interact with the core proteins of NF-kB to activate various auxiliary proteins (Visconte et al., 2019). The physical interaction of Smad3 and p52/RelB is known to activate Jun B expression (Luo, 2017). Brandl et al., 2010 demonstrated that TGF-β-SMAD signaling is regulated by IKKα by interacting with SMAD3 thereby governing SMAD complex formation on DNA. Furthermore, the TGF-β-IKKα-SMAD signaling downregulates E-cadherin and activates transcription of genes encoding Slug and Snail in pancreatic cancer cells. In addition, IKKα also modulates canonical TGF-β-SMAD signaling in human MDA-MB231 breast cancer cells thereby highlighting the impact of IKKα on TGF-β-SMAD signalling. (Brandl et al., 2010). In non-Smad pathway, TGF-β can also activate NF-kB by TGF-β-activated kinase 1 (TAK1). TAK1 activates IKK, which in turn phosphorylate IκBα, leading to proteasomal degradation of IKBα and the release of NF-kB p65-p50 heterodimer resulting in NF-κB activation. (Hydarpoor et al., 2020). Studies have demonstrated that TGF-TAK1 also induces NF-kB activation in murine B cells, hepatocytes and head and neck squamous cell carcinoma (HNSCC) cells (Loren et al., 2021). Freudlsperger et al. has demonstrated the aberrant TGF-TAK1 expression and its association with nuclear NF-κB activation in HNSCC tumors (Freudlsperger et al., 2013). In response to TGF-β, TAK one also activates RhoA-Rho-associated kinase (ROCK) resulting in the phosphorylation and activation of IKKβ, leading to NF-kB activation (Kwon et al., 2018a). In addition, TGF-β also evokes cellular response through activation of PI3K-Akt pathway leading to the phosphorylation of IKKα/β, IkB and NF-kB which results in increased integrin expression and cell migration (Kwon et al., 2018b). Thus, the key players in NF-κB signaling pathway not only function as signaling components but also can act as the crossroad between NF-kB and TGF-β pathways (Torrealba et al., 2019). Although, many studies suggest the role of TGF-β in activating NF-κB, repression of NF-κB signaling by TGF-β has also been reported (Zhang et al., 2020a). Several studies have suggested a critical role of inhibitory Smad, Smad7 in the crosstalk between TGF-β and NF-κB signaling (Chen et al., 2018b). The upregulation of Smad7 and its interaction with NF-κB subunit p65 suppresses TGF-β-Smad signaling (Ciceu et al., 2021). On the other hand, an increase in the expression of Smad7 can also induce IκBα, thereby inhibiting NF-κB activation (Lu et al., 2017). This inhibition of NF-kB by TGF-β could be attributed to the negative feedback loop (Lang et al., 2020). A study by Arsura et al. demonstrated that in murine B cells and hepatocytes, the initial activation of NF-kB leads to the transcriptional activation of IkB that eventually causes inhibition of NF-kB signaling (Wang et al., 2017). This feedback loop could act as an important target in attenuating the cytostatic response of TGF-β during malignant progression. Collectively, TGF-β signaling modulates NF-kB signaling and promotes transcriptional activity of various genes which are involved in cell proliferation, invasion, metastasis, EMT and associated inflammatory signaling to promote tumorigenesis. In conclusion, although some studies suggest that TGF-β is also regulated by IKK to prevent tumorigenesis, however, significant number of studies demonstrated that the cross talk of TGF-β-mediated Smad/NF-kβ drives transcription of tumorigenic genes for tumor cell proliferation, growth, invasion, angiogenesis and metastasis to distinct secondary sites (Figure 7).

### The JAK/STAT Signaling

**FIGURE 6:**
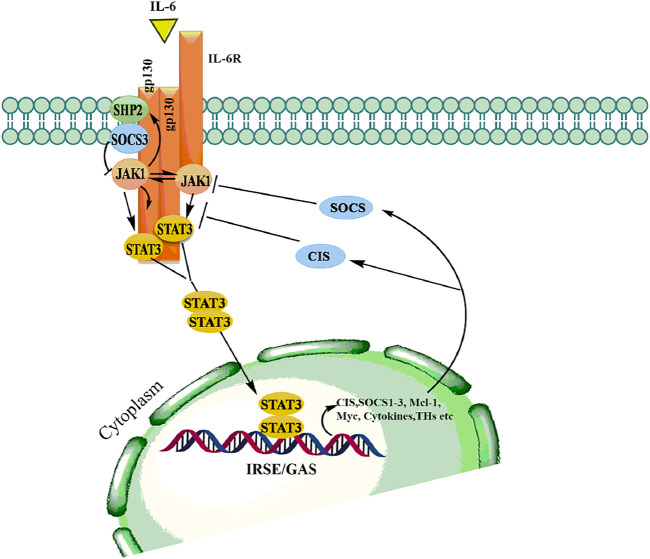
Binding of ligand to a cytokine receptor results in dimerization and conformational changes leading to activation of JAK, which in turn phosphorylates downstream mediator STATs thereby allows dimerization followed by translocation into the nucleus to modulate transcription various genes involved in hematopoiesis, immunity, growth and differentiation.

The JAK/STAT pathway mediates cellular responses to a wide array of cytokines and growth factors (Maude et al., 2015; Pencik et al., 2016). JAKs were initially named as “just another kinase”, but were later changed to “Janus kinase” which was attributed to being a unique class of tyrosine kinases that contain both a catalytic and kinase-like domain and possesses autoregulatory function (Genovese et al., 2017). Abundant evidence has supported the role of JAK/STAT in the regulation of various cellular processes including proliferation, differentiation, migration, apoptosis, and cell survival, depending on the signal, tissue, and cellular context (Pencik et al., 2016; Rios-Fuller et al., 2018). Mammalian JAK family contains four members: JAK1, JAK2, JAK3, and TYK2 each binding to different receptors. STAT family is composed of seven members STAT1, STAT2, STAT3, STAT4, STAT5a, STAT5b, STAT6, each having the tendency to bind to different cytokines (Siveen et al., 2018; Hammarén et al., 2019). The JAK proteins are relatively large kinases with more than 1,100 amino acids with a molecular mass between 120–130 KDa (Shahjahani et al., 2020). The JAK/STAT signaling is relatively simple and is activated by binding of extracellular ligands to the receptors that phosphorylated intracellular JAKs associated with them (Jan et al., 2021). Phosphorylated JAKs in turn create the docking site for downstream substrates, including both the receptor and the STATs (Bousoik and Montazeri Aliabadi, 2018). The activated STATs form homodimers in the cytoplasm followed by translocation to the nucleus where they bind to specific enhancer regions in target genes, thus regulating their transcription (Figure 6) (Hoi et al., 2016). Signal transducers and activators of transcription (STATs) belongs to a family of transcription factors, activated by Janus kinases (JAK) through phosphorylation of tyrosine residues in response to various cytokines and growth factors including macrophage colony-stimulating factor 1 (CSF-1), platelet-derived growth factor (PDGF), epidermal growth factor receptor (EGFR) (Singh et al., 2013) and interleukin-6 (IL-6) (Loh et al., 2019). The activated STAT3 forms a homodimers in the cytoplasm and transmits cytokine receptor generated signals by translocation into the nucleus (Mohassab et al., 2020). STAT3 binds to specific DNA response elements and regulates various processes that maintain the normal cellular homeostasis (Batista and Helguero, 2018).

The JAK/STAT signaling pathway plays a vital role in normal physiological processes. However, during the multistep process of carcinogenesis, JAK/STAT signaling pathway is persistently activated (Hu et al., 2020). Once in the nucleus, STAT3 homodimers binds to specific regulatory sequences and modulates the expression of many genes that have been shown to suppress apoptosis and induce cellular transformation (Abroun et al., 2015). Constitutive activation of JAK/STAT signaling has been implicated in various cancers including head and neck, gastric, breast, pancreatic, and prostate (Bose et al., 2020). Aberrant activation of JAK/STAT3 can mediate the recruitment of other molecules involved in tumorigenesis. STAT3 mediates its action by binding to the target genes involved in cell cycle regulation, cyclin D1 and inhibiting apoptosis by targeting anti-apoptotic Bcl-2, thereby contributing to cancer progression (Shao et al., 2021). The IL-6 associated JAK/STAT signaling pathway plays an important role in cancer development, and has proven to exhibit multifaceted properties to be considered as a therapeutic target for the treatment of cancer (Groner and von Manstein, 2017).

**FIGURE 7:**
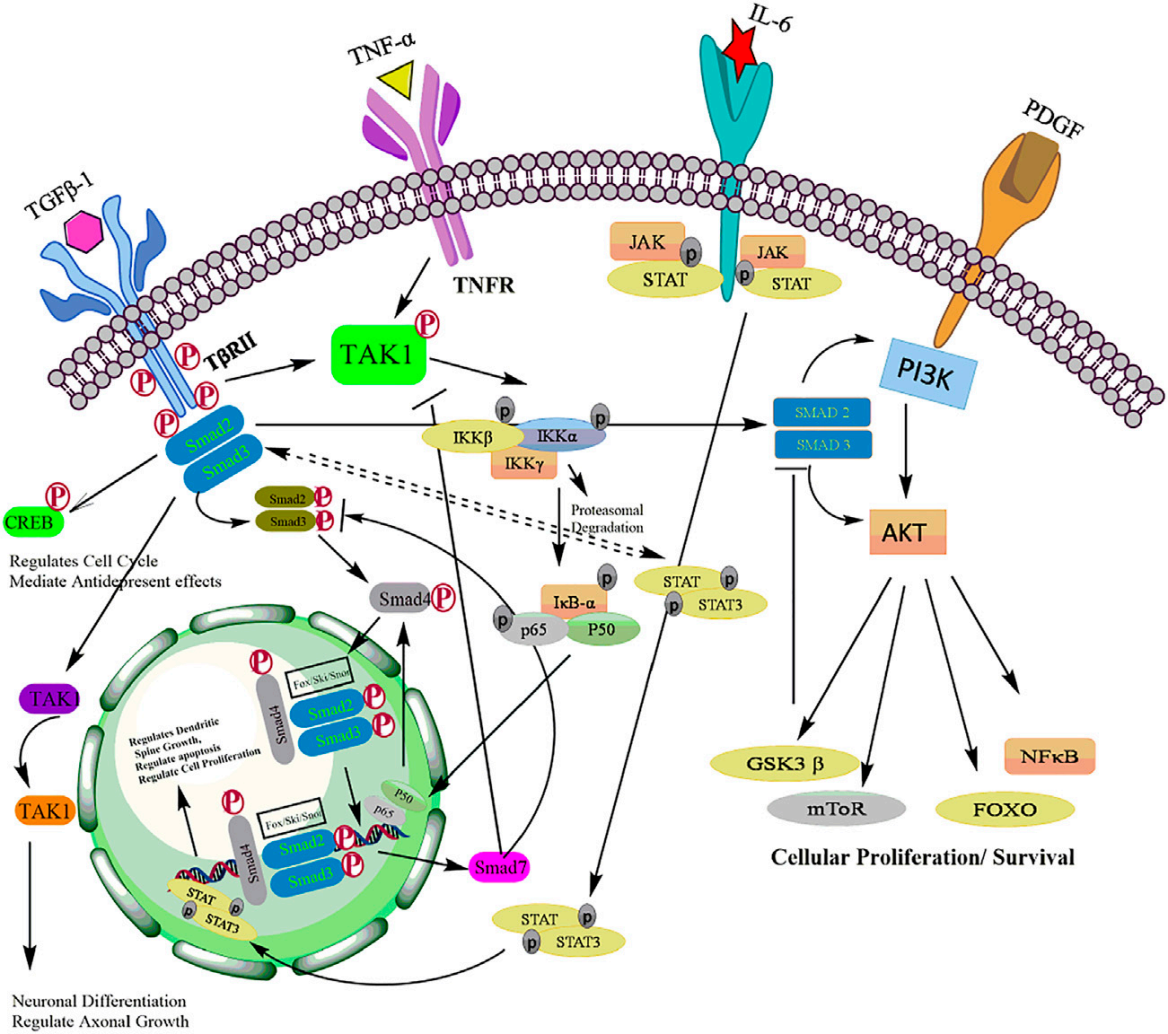
Crosstalk of TGF-β with other major signaling pathways including PI3K/Akt, NF-kB, and JAK/STAT signaling pathways.

Several reports have described the regulation of JAK-STAT by TGF-β in either positive or negative manner. Interleukin12-induced activation of JAK2 in T lymphocytes is inhibited by TGF-β resulting in inactivation of STAT3 and STAT4 (Salas et al., 2020). In contrary, TGF-β potentiates IL-6-induced STAT3 activation in hepatocytes and hematopoietic stem cells (HSC) (Rao et al., 2017). Moreover, it is also reported that TGF-β and Smad3 activation led to the elevated STAT3 phosphorylation in fibrosis and cirrhosis patient samples (Tang et al., 2017). The complex interaction between canonical Smads and STATS are highly involved in pluropotency and differentiation processes (Bertero et al., 2018). The Smad-mediated promoter activity requires Smad3/4 complex formation followed by nuclear translocation and activation of TGF-β responsive genes (Sakai et al., 2019). Conversely, STAT3 is known to interact with Smad3 to block Smad3/4 complex formation which attenuates the activity of TGF-β in inducing cell-cycle arrest and promoting EMT (Hao et al., 2019). JAK/STAT pathway indirectly regulates the activity of Smad3 by enhancing the expression of Smad7 (Syed, 2016). In human fibrosarcoma-derived cell line, INF-gamma induces expression of Smad7 mediated by phosphorylation and activation of the transcription factor STAT1 through JAK1 thereby preventing the interaction of Smad3 with TGF-β receptor (Majoros et al., 2017). Signal-transduction pathways induced by JAK/STAT and TGF-β signaling may be affected by transmodulating interactions between Smads and STATs (Chauhan et al., 2021). In conclusion, apart from regulating T-lymphocyte activation, TGF-β cross connects with JAK/STAT signaling to regulate plethora of pathophysiological process via Smads which includes activation of hematopoiesis, TGF-β fibrogenic responses in hepatic stellate cells, transcription of genes regulating EMT and regulating pluropotency and differentiations of cells (Figure 7).

## Conclusion

Recent advances in the molecular biology led to deep understanding in the areas of signaling networks and their role in cancer. Signaling cross-talk between different pathways orchestrates various cellular functions in an accurate, effective, and balanced manner. However, aberrant activation of these cellular signals and their targets could lead to catastrophic events. Although, the dual role of TGF-β signaling has been extensively studied in various biological processes including cancer, it may still appear to be complex. TGF-β signaling cross-talk is context dependent, and can be direct or indirect or a part of feed-back mechanism. The key players of TGF-β signaling and their interaction with other cellular networks play a decisive role in embryonic development, stem-cell renewal, differentiation and specify cell fate within the physiological context. With the identification of new interconnections and their targets, the TGF-β pathway has emerged as networking hub of cell signaling. Several studies with different approaches have provided clues about the versatility of TGF-β and its interactions with other signaling pathways. Some studies have shown the contradictory results to the established role the TGF-β signaling. This discrepancy could be due to the disparities in experimental conditions such as cell type, physiological/pathological status, developmental stage, localization of proteins, nature of modifying enzymes, co-factors etc. A future challenge for the researchers is to undergo in-depth mechanistic studies to identify the specific convergence point of these cellular pathways and to accurately predict biological outcomes. Recently, the role of TGF-β and associated signaling cascade has also been implicated in the regulation of microRNA, yet another unexplored area of TGF-β research. In addition, a number of studies have suggested the interconnection of TGF-β activity with energy metabolism (glucose uptake/consumption, AMPK and mTOR signaling) and NO (nitric oxide) signaling. The exciting progress in genome-wide mapping technologies and combinatorial approaches of therapies targeting the relevant signaling pathways along with the current techniques in genetics, molecular biology, and bioinformatics may reveal a detailed signaling network cascade and can also assist in elucidating the mechanism of the dual role of TGF-β, its functions and regulation under varying physiological contexts.
